# Occult Foreign Body of the Eyelid Presenting as Recurrent Eyelid Ecchymosis

**DOI:** 10.7759/cureus.15521

**Published:** 2021-06-08

**Authors:** Akshay G Nair, Amjad U Furniturewala

**Affiliations:** 1 Ophthalmic Plastic Surgery & Ocular Oncology, Advanced Eye Hospital & Institute, Mumbai, IND; 2 Ophthalmic Plastic Surgery & Ocular Oncology, Orbit Eye Hospital, Mumbai, IND; 3 Ophthalmic Plastic Surgery & Ocular Oncology, Aditya Jyot Eye Hospital, Mumbai, IND; 4 Cornea, Cataract and Refractive Surgery, Orbit Eye Hospital, Mumbai, IND

**Keywords:** ocular trauma, imaging modalities, high-resolution ct scan, oculoplastic orbital surgery, foreign bodies

## Abstract

Treatment strategies for treating periocular retained foreign bodies depend on the nature of the foreign body, its composition, size, location, and presenting symptoms. Foreign bodies retained in the ocular adnexa can be asymptomatic and lie dormant for long periods of time. In this communication, we present the case of a 32-year-old female who presented with a history of multiple episodes of recurrent edema and ecchymosis of the left lower eyelid, occurring over the past three years. She had been involved in a vehicular accident 13 years ago, which resulted in multiple facial lacerations. She subsequently underwent primary wound repair and two skin grafting procedures. Imaging revealed a hyperdense foreign body located just within the inferolateral orbital rim. An exploration was performed, and a glass foreign body was recovered. We hypothesize that the dormant foreign body had migrated, and repeated microtrauma caused by the sharp edges of the glass piece, either spontaneous or triggered by trivial trauma such as eye rubbing, led to episodes of eyelid hemorrhage and edema. The unique aspects of this case are the unusually long period of quiescence before which the symptoms appeared, the atypical clinical signs, and the eventual recovery of this occult foreign body from the eyelid. This case also underscores the importance of a detailed history and the need for imaging in facial trauma.

## Introduction

Periocular foreign bodies can be classified as organic or inorganic. Some foreign bodies cause complications, whereas others are asymptomatic and remain undetected for months or years [1]. Organic foreign bodies like wood usually cause an inflammatory reaction that is typically characterized by the formation of granulation tissue and, in some cases, infection. Nonorganic materials like glass, plastic, gold, and silver are inert; however, other nonorganic metallic foreign bodies can be toxic and tend to elicit a local irritative response, suppuration, or specific degenerative effects. Local irritation leads to fibrous tissue proliferation and encapsulation of the foreign body. In this case, the recovery of a hidden glass foreign body from the eyelid is reported. It is believed that this foreign body had been present for 13 years and possibly migrated over time. The present case illustrates the need for careful history taking and imaging in the setting of a previously repaired trauma presenting with unusual symptoms.

## Case presentation

A 32-year-old female presented with complaints of multiple, recurrent edema and ecchymosis of the left lower eyelid. The episodes, which first started three years prior to presentation, were spontaneous, and the subsequent swelling and discoloration were self-resolving. The patient also noticed one episode in the past when the ecchymosis developed after an episode of eye-rubbing. The patient had been in a vehicular accident involving a windshield injury 13 years prior. She had been operated upon immediately following the trauma and underwent two subsequent skin grafting procedures, the last being five years prior to presentation. On examination, the visual acuity in both eyes was 20/20 N6. The left eyebrow had focal loss of hair with scarring. The left lower lid had a shallow notch with some scarring over the skin. A dark, ecchymotic patch was seen involving the lateral half of the left lower lid extending downwards from the eyelid margin, measuring approximately 2 cm × 2 cm. Anterior and posterior segment evaluation of both eyes was unremarkable with normal intraocular pressure.

**Figure 1:**
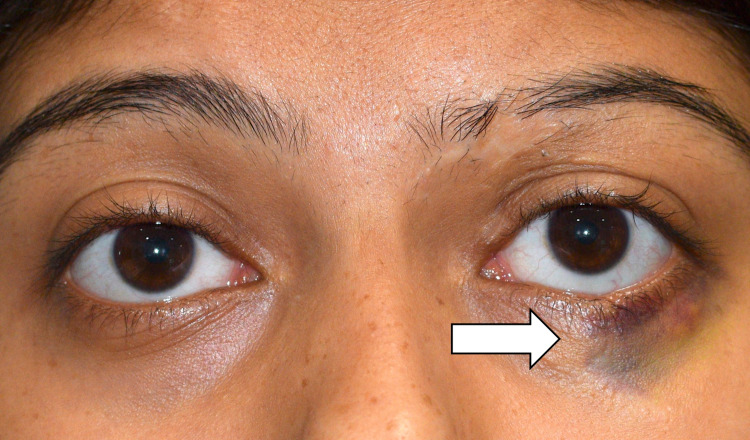
External photograph of the patient highlighting the ecchymosis involving the left lower lid (white arrow).

**Figure 2:**
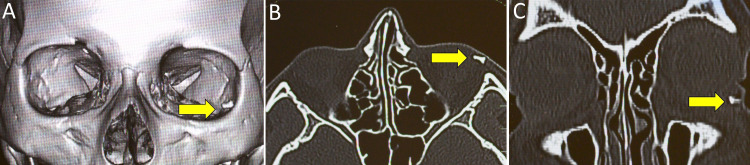
Three-dimensional reconstructed CT scan image showing the location (white arrow) of the foreign body with respect to the orbital rim (A). The sharp edges can be appreciated in the axial scan (B) and the coronal slice (C). CT: computed tomography

**Figure 3:**
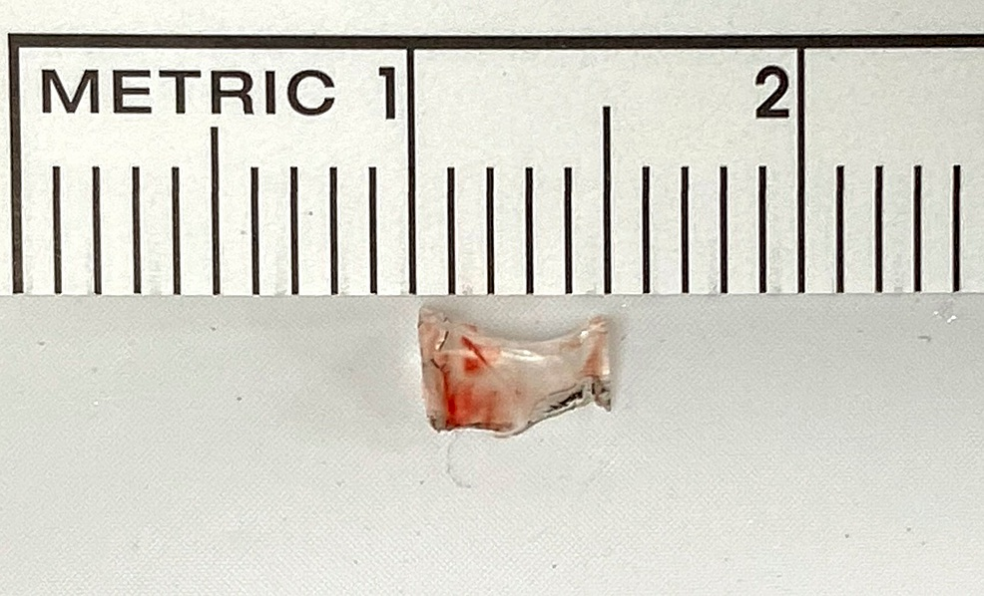
The retrieved glass foreign body measuring 3 mm × 5 mm.

The patient’s recovery was uneventful with no recurrences of the swelling or ecchymosis. The patient had no measurable proptosis or enophthalmos and ocular motility was normal in both eyes. The orbital rims were normal on palpation and the eyelid skin showed no change on asking the patient to perform the Valsalva maneuver. On lid eversion, the conjunctiva was healthy with no visible scarring. CT of the orbits was performed, and the scans showed an unidentified, hyperdense object, measuring about 0.5 cm × 0.5 cm, with sharply defined edges sitting just within the left inferotemporal orbital rim (Figure 2). On deep palpation, which elicited sharp pain, a small, hard, nodular mass was palpable. A diagnosis of an occult foreign body was made, and exploration was carried out through a skin incision which was made over the presumed location of the foreign body based on the scans. Although the option of a transconjunctival route was available, a skin incision was made over the preexisting scar. Under the orbicularis oculi muscle and deep within the lateral fat pad of the lower eyelid, unencapsulated, sharp glass shrapnel was found and removed in toto. The dimensions of the mass matched the radiological measurements and the wound was closed in layers (Figure 3).

## Discussion

Some foreign bodies that are retained within the orbit and eyelid can cause immediate complications, whereas others are asymptomatic and remain undetected for long periods of time. Therefore, a detailed history is crucial in patients and should include specific questions about antecedent trauma and its details, especially in patients with soft tissue complaints.

Carrol reported a similar case of a patient who had presented with repeated episodes of pain and subconjunctival hemorrhage. A recent finding was a small, mobile, nodular swelling that was palpable over the eyelid. A glass foreign body was retrieved on exploration, which was embedded in the eyelid tissue 28 years previously at the time of an automobile accident. It most likely began to change position after 27 years when the first subconjunctival hemorrhage occurred [2].

In our patient, the initial trauma was a windshield injury, 13 years prior to presentation. Multiple cuts to the upper third of the face along with glass pieces embedded within the soft tissue are a common feature of windshield facial injuries [3]. It is likely that the glass piece may have lodged itself during the trauma and stayed undiscovered in the absence of any form of imaging having been performed. It has been known that soft tissue foreign bodies that were missed on initial evaluation may migrate later, giving rise to new symptoms. This can occur months or years after the traumatic event [1]. There have been many proposed mechanisms that can lead to migration of a foreign body like tissue restitution, the effect of gravity, and rotation or movement of the retained particle due to its intrinsic property or sharpness [4]. In such cases, ultrasound, CT scans, and MRI are useful to identify radiolucent foreign bodies. In our case, the foreign body was a glass piece. Glass is always radiopaque, and its radiopacity does not depend on its lead content or other metal content [5]. By virtue of being an inert, inorganic foreign body, glass does not elicit an inflammatory reaction. Encapsulation or fibrous capsule formation around glass has not been reported. The repeated edema and bleeding resulting in ecchymosis over the eyelid was due to the glass piece rubbing against structures in the vascular lower lid following its migration.

## Conclusions

To summarize, we present a rare case of a glass foreign body embedded in the eyelid 13 years prior to presentation. The only clinical manifestation, in our case, was recurrent eyelid edema and ecchymosis. Imaging at the time of trauma and primary repair, even in cases where only soft tissue appears to be involved, can help in identifying any foreign bodies. Migration of the foreign body, may give rise to symptoms, years after the initial trauma.
